# Pretibial Full Thickness Skin Burn following Indirect Contact from Bone-Cement Use in a Giant Cell Tumour

**DOI:** 10.1155/2007/81592

**Published:** 2007-12-09

**Authors:** Buchi Rajendra Babu Arumilli, Ashok Samuel Paul

**Affiliations:** The Regional Sarcoma Group, Department of Orthopaedics & Trauma, Manchester Royal Infirmary, Oxford Road, Manchester M13 9WL, UK

## Abstract

Bone cement reaches significant temperatures and is known to cause thermal and chemical damage to various tissues. All the reports of such damage occurred following a direct contact of the tissue or structure with cement. We report the case of a patient with a giant cell tumour of the proximal tibia who underwent curettage and bone cement application through a posterior approach and subsequently developed full thickness pretibial skin damage despite showing no evidence of any direct contact of the involved skin with bone cement. This is the first report of its kind and though anecdotal is a serious complication that surgeons should be aware of.

## 1. INTRODUCTION

Bone cement is used commonly in various subspecialities of orthopaedics. The thermal effects of bone cement on various tissues like nerves [1], vessels [2], bladder [3], and the bone [4] after direct contact have been reported. Skin damage from bone-cement use has been previously reported but was either following cement extrusion from a cortical defect [5] or skin contact with discarded cement [6]. Aggressive curettage and cement application are a procedure done with reasonable success for large giant cell tumours of bone [7]. We report the case of a patient who developed full thickness skin damage after curettage and bone cement application for a giant cell tumour of the proximal tibia despite there being no evidence of a direct contact.

## 2. CASE REPORT

A 31 year old, male patient was referred to us for the follow-up care of his left proximal tibial giant cell tumour. He underwent extensive curettage and bone cement application at an orthopaedic unit ten days before, which was undertaken through a posterior approach after making a cortical window. A tourniquet was used, patient was positioned prone with good padding over the bony prominences and no other adjuvant was used in the procedure except bone cement. The procedure lasted for 55 minutes and there were no intraoperative concerns. Within the first postoperative day, a blister measuring 5 × 3 cm was noted over the anterior aspect of the knee just medial to the tibial tubercle.

At 4 weeks after the index procedure, the blister turned into a well-defined eschar measuring 6 × 4 cm. Although initial management was nonoperative, patient was informed about the possibility of débridement. After five weeks, excision of the area and secondary grafting was needed as no further signs of healing were evident. Intraoperative damage involved the whole thickness of the skin and subcutaneous tissues. The base of the eschar was the tibial periosteum, there was no macroscopic evidence of a cortical breach or cement extrusion underneath. Culture swabs from the deep tissues failed to reveal any infection. The area healed at the end of 8 weeks after the initial surgery without further intervention.

## 3. DISCUSSION

Giant cell tumours are often large, juxta-articular lesions with a significant rate of local recurrence. Bone-cement use in managing these tumours has a dual advantage of providing good structural integrity along with the potential tumoricidal effect [7]. The main disadvantages highlighted in the literature of using cement in giant cell tumours are the potential damage to the articular cartilage [7] and the development of a radiolucent zone at the bone cement interface [8].

The effects of bone cement on normal tissues have been studied extensively in vitro and to some extent in vivo. PMMA (bone cement) causes thermal necrosis due to the high heat of polymerisation and chemical necrosis due to the unreacted polymer [9]. Thermal necrosis has been reported in bone after exposure to 50 deg C for more than one minute [9]. The surface temperature of setting cement mantle which is 10 mm thick could reach 107 deg C at room temperature [10]. Bone necrosis has been proved histologically up to a depth of 2 mm from the surface when cement mantles of 3 mm thick were used in models simulating knee arthroplasty [11] and also in animal studies simulating giant cell tumour surgery [12, 13]. Such damage to the bone is proportional to the volume of cement used [14].

Connective tissues responded to heat by showing chronic damage histologically after exposure to temperatures from 43 deg C which increased with dose [15]. The diffusion of heat by soft tissues increased by 10% when the tissues were preheated to 75 deg C [16]. Studies in cadavers [17] and laboratory [18] have investigated on temperature increments at varying distances from bone cavities (1, 2, 3, 5, and 10 mm) after bone-cement use. Temperatures reached between 30–40 deg C at a distance of 3 mm from the cavity surface when a cavity of 40 centimetre cube (3) volume was filled with cement [16]. Although it is difficult to quantify temperatures in vivo, nonetheless when large volumes of cement (double mix of 80 g) are used as in tumour surgery, theoretically, enough heat could be generated affecting the surrounding soft tissues.

In this patient case, the proximity of the skin and subcutaneous tissues to the anterior part of proximal tibia lead to a significant full thickness damage due to thermal conductivity from the underlying bone. Two previous reports of skin burn due to bone-cement use have been found in the literature. One report was following subcutaneous cement extrusion following a revision total knee replacement [5] and the other was following a prolonged contact of skin with a piece of discarded cement during a total hip replacement [6]. The possibility of a pressure sore in our patient is unlikely as the presentation (blister turning to an eschar) was similar to the two reports in the literature. Thus, this is the first report so far of a spontaneous full thickness skin damage following the use of large volumes of bone cement without evidence of a direct contact between the two as radiologically no cortical breach was noted and intraoperatively no cement extrusion found.

The more important implication of such damage to soft tissues is when an anterior approach is used. There might be noncontact thermal necrosis of soft tissues posterior to the intact proximal tibia that might go undetected and could cause catastrophic neurovascular complications. We stress the importance of warning patients of such complications when consenting especially for procedures where large volumes of bone cement might be used.

## 4. CONFLICT OF INTERREST STATEMENT

No benefits of any kind have been or will be received by the authors for the preparation or publication of this report.

## Figures and Tables

**Figure 1:**
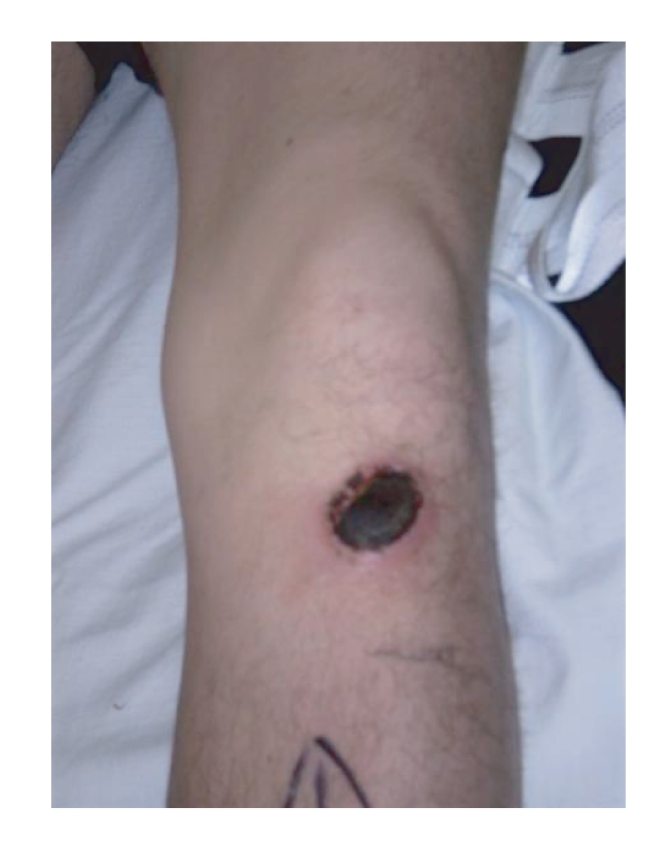
Skin burn over the anterior aspect of left leg.

**Figure 2:**
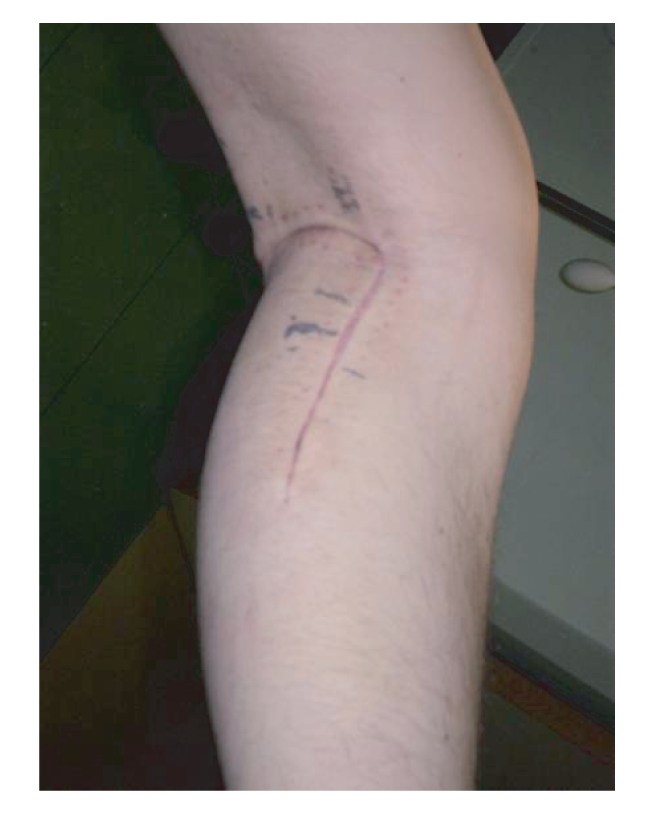
The posterior approach used for index operation (curettage and bone cement application) on left leg.

**Figure 3:**
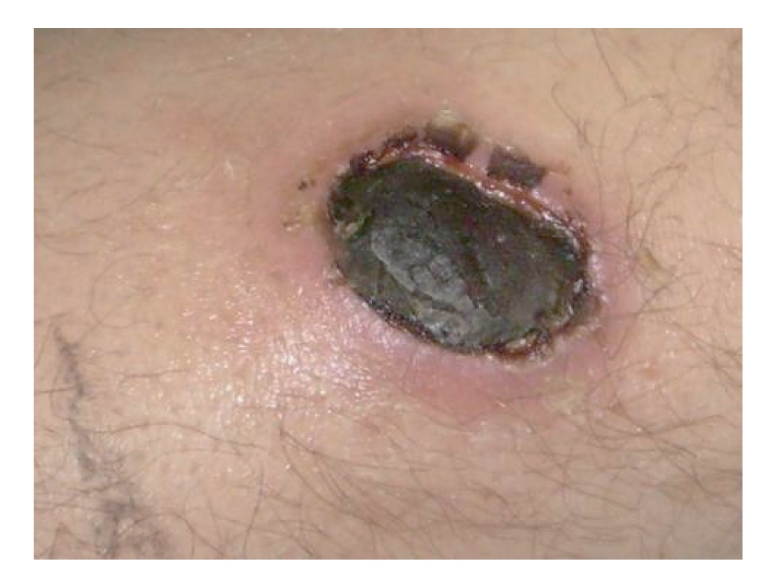
Close-up view of the eschar on the left pretibial area.

**Figure 4:**
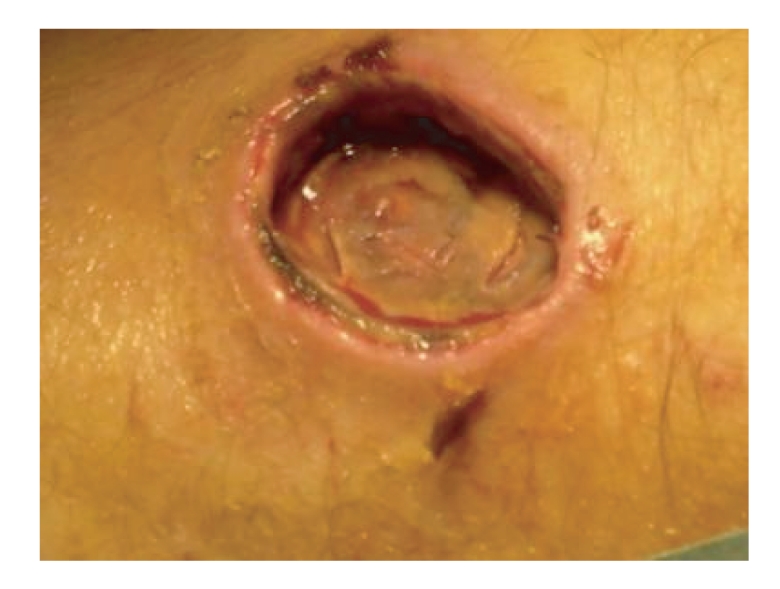
Intraoperative picture following débridement of the eschar showing the full thickness skin damage without any cement extrusion underneath.

**Figure 5:**
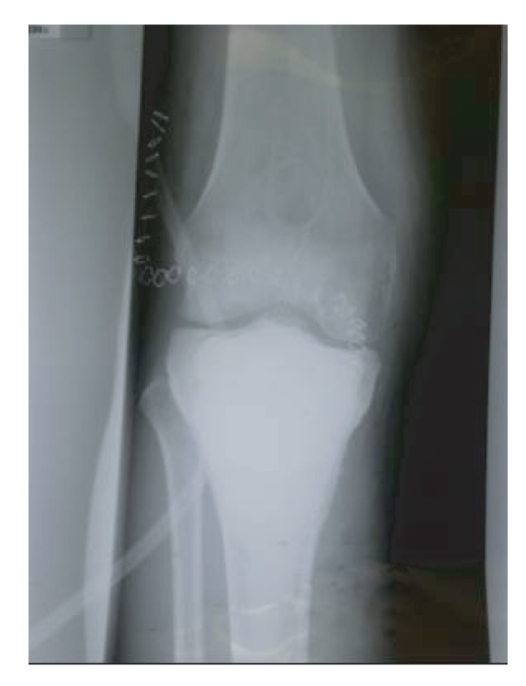
Postoperative AP view of the left knee.

**Figure 6:**
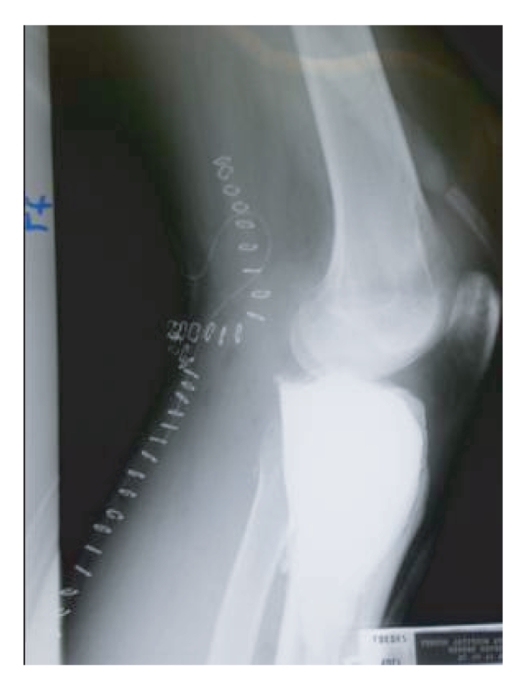
Postoperative lateral view of left knee showing no cortical breach/subcutaneous cement extrusion anteriorly.
